# Developmental Immunotoxicity Testing and Protection of Children's Health

**DOI:** 10.1371/journal.pmed.0030296

**Published:** 2006-08-22

**Authors:** Rodney R Dietert

## Abstract

Dietert discusses a new study in *PLoS Medicine,* by Heilmann et al., showing that children exposed to polychlorinated biphenyls (PCBs) have reduced antibody responses to childhood vaccinations.

Prenatal and postnatal exposure to persistent organochlorine pollutants, such as polychlorinated biphenyls (PCBs), has been implicated as a possible cause of impaired immune function in children. For example, studies have shown that children who have been exposed to PCBs and related compounds from their mothers' contaminated diets have reduced concentrations of immunoglobulins and increased frequency of childhood infections [1–3]. And a new study by Heilmann et al. published in *PLoS Medicine* shows that children exposed to PCBs have reduced antibody responses to childhood vaccinations, adding to the growing evidence that PCBs are associated with developmental immunotoxicity (DIT) [4]. The study provides a stark example of the heightened immune vulnerabilities that exist during early life and that must be adequately protected from environmental insult if we are to minimize health risks to children.

## The New Study

In their prospective epidemiological study, Heilmann and colleagues studied two birth cohorts from the Faroe Islands. These islands are a unique setting for studies of PCB immunotoxicity because average PCB exposures are up to 10-fold higher than average levels in Northern Europe, due to the traditional habit of eating pilot whale blubber. Heilmann et al. assessed PCB exposure perinatally by using quantitation of major PCB congeners (components) in pregnancy serum, transition milk (produced immediately following colostrum secretion), and children's serum. Following routine childhood vaccinations with two T cell–dependent vaccines (tetanus and diphtheria), children were examined at 18 months and at seven years, and serum was analyzed for tetanus and diphtheria toxoid antibodies and for PCBs. The researchers found that prenatal and perinatal PCB levels were excellent predictors of antibody responses to the vaccines (for example, antibody response to diphtheria toxoid decreased at age 18 months by 24.4 percent [95 percent confidence interval, 1.63–41.9] for each doubling of the cumulated PCB exposure at the time of examination).

Children exposed to PCBs have reduced antibody responses to childhood vaccinations.

It is interesting that early-exposure PCB levels were better predictors of antibody response to vaccines at seven years of age than were contemporary PCB levels, despite the capacity of PCBs to be retained in fatty tissues. This suggests that even transitory early-life exposures to an immunotoxicant have the potential to produce immune problems in later life. Furthermore, one should not assume that toxicant body burdens determined at the time of immune assessment represent effective correlates for DIT.

In Heilmann and colleagues' study, PCB exposure was negatively associated with antibody responses to the immunizations. For example, even after receiving a booster vaccination, more than 20 percent of the children from one cohort had antibody titers to diphtheria below the level needed for long-term protection. Based on the results, the authors suggested that even low- to moderate-level PCB exposures are likely to influence childhood immunization responses. This is consistent with the findings of Marchant et al. [5], who showed that environmental factors contribute significantly to the persistence and avidity maturation (increased antibody binding strength) of antibody responses during childhood vaccine responses.

## Implications of the Study

The study results have three important implications. First, Heilmann et al. used a very valuable measure of DIT, namely, a T cell–dependent functional immune response [6]. Using this response is valuable because immunotoxicity in early life can result in major shifts in immune function capacity without necessarily producing profound loss or alteration of immune cell populations [7,8]. For this reason, we should be cautious about depending on circulating leukocyte profiles to detect DIT. Heilmann and colleagues' use of functional immune measures is to be applauded and should be used whenever possible in assessing early-life immunotoxicity. In a recent review on the topic, Luster et al. [9] discussed the challenges of detecting subtle, yet functionally important, immune alterations in children. These authors suggested that the optimum methodology for detecting DIT in humans would include an evaluation of functional responses to childhood vaccinations. Therefore, Heilmann and colleagues' study serves as a prototype that will hopefully lead to other studies of the relationship between exposure to chemicals and drugs and childhood immunization responses.

A second implication of the study is that there are likely to be more examples of environmental exposure to drugs and/or chemicals causing immune dysfunction and/or targeted immunosuppression across a population than examples of such exposure causing profound immunodeficiency. In the era of AIDS as a prototype example of immunosuppression, we have tended toward the default assumption that immunosuppression is associated with readily detectable losses of circulating immune cells. But this is probably the exception rather than the rule. For example, even with the profound loss of cell-mediated immunity associated with heavy metal–induced Th1-dependent immunosuppression, only minimal changes in immune cell populations are detectable [7]. Ineffective responses to vaccines affecting particular segments of the population and/or immune-associated increases in the incidence of allergic disease or asthma are current public health issues [10,11], and we must identify causative environmental agents associated with such changes. But prior to the current study by Heilmann et al., we have had far too few studies designed with prospective assessment that included the necessary functional measures to detect associations between exposure to environmental toxins and immune dysfunction.

A third ramification of the study's results applies more broadly to current regulatory approaches for protecting children's health. Recent studies established that the developing immune system is a far more sensitive target for immunotoxicity than that of the adult [7,8,12]. In fact, for PCB-related dioxin exposure in rodents, there appears to be an approximately 100-fold difference in dose sensitivity across age groups [12]. Studies conducted to date show that early-life stages are sensitive to far lower levels of immunotoxicants than adults, and the toxic effects are more extensive and persistent following perinatal exposure than adult exposure. Additionally, toxicity testing using adults is ineffective in predicting the immunotoxic risk to children following early-life exposure [8]. This realization occurs at a time when we have witnessed recent increases in immune-related childhood diseases such as asthma [13]. Recent consensus workshops have attributed the increased incidence of childhood asthma largely to environmental causes [11,14], and, in turn, environmental factors that are protective during early life have also been identified through recent research [15].

## Protecting Children's Health

Regrettably, these research advances have not yet been applied to improved regulatory protection of children's health. This gap between research results and actual health protection needs to be closed. DIT testing is not currently required to ensure that gestational and neonatal exposure to drugs and chemicals is safe for the child's immune system. Some clinicians and parents are probably unaware that most previously approved drugs and chemicals were never tested for developmental immunotoxic potential, and that those currently under consideration for approval are not required to be tested based on recent guidelines [16]. In light of the current findings [4], as well as studies showing the vulnerability of the developing immune system [12], it seems time to reconsider the wisdom of exposing pregnant women, infants, and children to drugs and chemicals with unknown developmental immunotoxic risks.

## Abbreviations

DIT: developmental immunotoxicity
PCB: polychlorinated biphenyl
